# An overall survival predictive nomogram to identify high-risk patients among locoregionally advanced nasopharyngeal carcinoma: Developed based on the SEER database and validated institutionally

**DOI:** 10.3389/fonc.2023.1083713

**Published:** 2023-03-16

**Authors:** Yinbing Lin, Jiechen Chen, Xiao Wang, Sijie Chen, Yizhou Yang, Yingji Hong, Zhixiong Lin, Zhining Yang

**Affiliations:** ^1^ Department of Radiation Oncology, Shantou University Medical College Cancer Hospital, Shantou University, Shantou, China; ^2^ Shantou University Medical College, Shantou University, Shantou, China; ^3^ Nasopharyngeal Carcinoma Research Center, Shantou University Medical College Cancer Hospital, Shantou, China

**Keywords:** head and neck cancer, nasopharyngeal carcinoma, locoregionally advanced, SEER database, nomogram, overall survival, high risk

## Abstract

**Objective:**

Locoregionally advanced nasopharyngeal carcinoma (LA-NPC) patients, even at the same stage, have different prognoses. We aim to construct a prognostic nomogram for predicting the overall survival (OS) to identify the high-risk LA-NPC patients.

**Materials and methods:**

Histologically diagnosed WHO type II and type III LA-NPC patients in the Surveillance, Epidemiology, and End Results (SEER) database were enrolled as the training cohort (n= 421), and LA-NPC patients from Shantou University Medical College Cancer Hospital (SUMCCH) served as the external validation cohort (n= 763). Variables were determined in the training cohort through Cox regression to form a prognostic OS nomogram, which was verified in the validation cohort, and compared with traditional clinical staging using the concordance index (C-index), Kaplan–Meier curves, calibration curves and decision curve analysis (DCA). Patients with scores higher than the specific cut-off value determined by the nomogram were defined as high-risk patients. Subgroup analyses and high-risk group determinants were explored.

**Results:**

Our nomogram had a higher C-index than the traditional clinical staging method (0.67 vs. 0.60, p<0.001). Good agreement between the nomogram-predicted and actual survival were shown in the calibration curves and DCA, indicating a clinical benefit of the nomogram. High-risk patients identified by our nomogram had worse prognosis than the other groups, with a 5-year overall survival (OS) of 60.4%. Elderly patients at advanced stage and without chemotherapy had a tendency for high risk than the other patients.

**Conclusions:**

Our OS predictive nomogram for LA-NPC patients is reliable to identify high-risk patients.

## 1 Introduction

Nasopharyngeal carcinoma (NPC) is a relatively uncommon malignant tumor with extremely unbalanced geographical global distribution (1). There were about 133,354 new cases of NPC in 2020 according to the International Agency for Research on Cancer, of which >70% were in east and southeast Asia (1, 2). Unfortunately, since early nasopharyngeal carcinoma is relatively asymptomatic, at diagnosis, more than 70% of NPC cases are classified as locoregionally advanced disease (LA-NPC: stage III-IVB in editions of TNM staging system before 2016; stage III-IVA after the 2016 edition) (3, 4), which has markedly unsatisfactory prognosis compared to early stage (3). However, even though LA-NPC patients are at the same TNM stages and are treated with identical or similar regimens, they still have diverse survival rates, with approximately 30-40% eventually developing distant metastasis after undergoing radical treatment (5, 6). Due to inherent individual heterogeneity, the traditional TNM staging system has limited accuracy in prognosis prediction. An increasing number of approaches have been used to develop improved predictive models to identify higher-risk and lower-risk LA-NPC populations for the purpose of trimming treatment strategy so as to achieve better outcomes and/or to reduce treatment side-effects. Construction of a nomogram is one of the most popular research directions (7–15). By integrating variables identified to have a significant impact on outcome, nomograms are excellent visualization tools for the estimation of survival rates, but also identify the critical parameters responsible for survival (15). Reported studies examining into some specific subgroups of NPC, including some tumor stage (16–22), patient age (20, 23–26), histologic type (27–29) and even race (30–32), in a wide scope based on the Surveillance, Epidemiology, and End Results (SEER) data, have shown the potential and ability to provide a better reference for clinical decisions (16, 21, 23). However, few studies have specifically focused on LA-NPC patients as a group using the SEER data. In this study, we develop a prognostic nomogram to predict the survival of LA-NPC patients by using the SEER database, and validate the nomogram with the patient data of our institution. Ultimately, we acquired a nomogram capable of better identifying high-risk LA-NPC patients than traditional TNM staging.

## 2 Materials and methods

### 2.1 Patient selection

Data of 6738 NPC cases was downloaded from SEER*Stat software, version 8.4.1 and rooted from the Incidence-SEER Research Plus Data, 17 Registries, Nov 2021 Sub (2000–2019). Selection statement: (Site recode ICD-O-3/WHO 2008 = “Nasopharynx”) AND (Behavior code ICD-O-3 = “Malignant”) AND (Diagnostic confirmation = “Microscopically confirmed”) AND (Histology recode - broad groupings = “8050-8089: squamous cell neoplasms”). The inclusion criteria were as follows (1): histopathologically confirmed NPC (2); histological subtypes of World Health Organization (WHO) type II nonkeratinizing squamous cell carcinoma (NKC, 8072, 8073) and WHO type III basaloid squamous cell carcinoma (BSCC, 8083) categorized by the International Classification of Diseases of Oncology, Third Edition (ICD-O-3) (3); stage III, IVA, IVB according to the 7th edition of the American Joint Committee on Cancer (AJCC) staging system; and (4) received radiotherapy. There were 421 eligible LA-NPC cases, which served as our training cohort. An additional set of 763 consecutive LA-NPC patients, treated between 2012-2014 in Shantou University Medical College Cancer Hospital (SUMCCH), were used to establish the external validation cohort.

### 2.2 Variable collection

Clinical variables were collected in both cohorts: gender, age, pathological subtypes according to the WHO, tumor (T) stage, node (N) stage and clinical stage according to AJCC (7th edition), chemotherapy, overall survival (OS) and survival status. For the external validation cohort, we collected clinical information of patients with LA-NPC (7th AJCC stage III-IVB) attending SUMCCH, who were then restaged using magnetic resonance imaging, according to the 8th edition of the AJCC staging system (7^th^ edition: III-IVB = 8^th^ edition: III-IVA). After re-staging, 758 patients with LA-NPC were selected for further analysis. Clinical factors in the two datasets were compared using the independent samples t-test for continuous variables and chi squared test for categorical variables. The continuous variable age was converted into an ordinal categorical variable using X-Tile software (Version 3.6.1, Yale University) for determining the optimal cut-off value (33).

### 2.3 Model development

The univariate Cox regression algorithm was used for the training cohort to analyze clinicopathological predictors of OS, and predictors with *p*-values ≤0.15 were selected for multivariate Cox analysis (enter selection). Finally, our nomogram for predicting OS at the 1-, 3- and 5-year stage was constructed using the function nomogram(), by which each patient’s corresponding total point was calculated.

### 2.4 Model evaluation & comparison

Internal 1,000-times bootstrap resampling was performed to evaluate the performance and optimism of the developed model according to the Transport Reporting of a Multivariable Prediction Model for Individual Prognosis of Diagnosis (TRIPOD) statement (34). The OS prediction model was verified using the external validation cohort. In the validation cohort, the model was also tested using the 8th edition of the AJCC/UICC staging system for staging patients. The discrimination, calibration and clinical effectiveness of the prediction model were metrices to determine the performance of the model (15) and was compared with the traditional TNM staging system. The discriminative ability of the predictive model was measured by the bias-adjusted C-Index (“rms” package) with its 95% confidence interval (CI) between which the ‘‘compareC” package and the function of cindex() in the “pec” package were used to quantify and visualize the differences (15). Patients were sorted by values of their corresponding total points, after which we used the function of quantile() to produce 2 cut-off values and stratify patients into high-risk, intermediate-risk and low-risk groups. The discriminative ability of the predictive model was measured by the adjusted C-index as described above (15). Kaplan–Meier survival curves showing the different risk subgroups were compared using a log-rank test. To visualize the OS of actual occurrence versus nomogram prediction, the function of calibrate() was used to construct the calibration plot (15). Decision curve analysis (DCA), in the ggDCA package, was used to assess the clinical utility of the predictive model (15, 35).

### 2.5 Subgroup analysis

The nomogram was validated for the subgroups of stage III and stage IV patients from the external cohort. Subtypes of stage III and stage IV were regarded as predictive factors. C-indices and risk stratification ability were compared between the nomogram and the subtypes.

### 2.6 Identification of prognostic factors for the high-risk group

To make the factors easier to analyze at a glance, we made scatter plots for visual representation of the distribution of the high-risk patients among different categories. We performed multivariate logistic regression analysis to investigate the independent influencing factors of high-risk patients in the validation cohort and Dominance analysis was then used to evaluate the relative importance of independent predictors.

### 2.7 Statistical analysis

**Figure 1:**
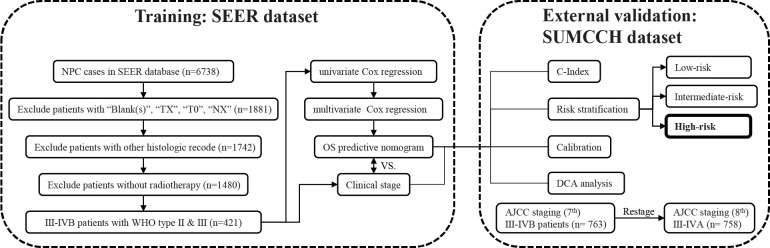
The entire analysis procedure. SEER, Surveillance, Epidemiology, and End Results; SUMCCH, Shantou University Medical College Cancer Hospital; NPC, nasopharyngeal carcinoma; WHO, World Health Organization; OS, overall survival; C-index, concordance index; DCA, decision curve analysis.

R software (version 4.2.0) and SPSS (version 20.0) were used for the statistical analysis. We regarded metrices with *p*-values <0.05 (two sided) as having statistical significance. An overview of the research design is shown in **Figure 1**.

## 3 Results

### 3.1 Patient baseline characteristics

The median follow-up for the SEER dataset was 59 months (range 1-119) and 169 deaths (40.1%) were observed. The median follow-up for the SUMCCH patients was 89 months (range: 3-117) and 205 deaths (26.9%) were observed. Patient demographic and clinical characteristics of the two datasets are illustrated in **Table 1**. All characteristics except gender and chemotherapy were significantly different between the two datasets.

**Table 1:** Clinicopathologic characteristics of the LA-NPC patients in the training and validation cohorts.

Variable	Training	Validation	*P*-value^b^	Restaged	*P*-value^b^
	cohort	cohort^a^		cohort^c^	
Age			<0.001		<0.001
<=52	195	452		451	
52-63	137	237		233	
>=64	89	74		74	
Sex			0.566		0.55
Female	121	206		204	
Male	300	557		554	
Histologic type			0.699		0.68
NKC	405	729		724	
BSCC	16	34		34	
T stage			<0.001		<0.001
T1	90	42		42	
T2	61	126		139	
T3	115	413		416	
T4	155	182		161	
N stage			<0.001		<0.001
N0	52	37		37	
N1	92	178		173	
N2	192	442		443	
N3	85	106		105	
Clinical stage			<0.001		<0.001
III	198	491		506	
IVA	138	166		252	
IVB	85	106		0	
Chemotherapy			0.485		0.65
Yes	21	47		44	
No	400	716		714	

NKC, nonkeratinizing squamous cell carcinoma; BSCC, basaloid squamous cell carcinoma; T, tumor; N node.

a Patients were diagnosed according to the 7th edition of the American Joint Committee on Cancer (AJCC) staging system.

b P-values were calculated with the chi-square test in the categorical variables between the cohort and the training cohort.

c Patients were restaged using magnetic resonance imaging according to the 8th edition of the AJCC staging system.

### 3.2 Univariate & multivariate analysis of the training cohort

Univariate Cox analysis indicated that clinical variables age, T stage, N stage and chemotherapy were associated with OS (*p ≤* 0.10). These significant clinical characteristics were included in multivariate Cox analysis, which further confirmed them to be critical factors influencing OS (**Table 2**). Older patients and those with more advanced T stage had poorer prognosis, whereas patients who underwent chemotherapy seemed more likely to have better outcomes.

**Table 2:** Univariate and multivariate Cox analyses of LA-NPC patients.

Variable	Univariate analysis		*P*-value	Multivariate analysis		*P*-value
	HR	95% CI		HR	95% CI	
Age						
<=51	Reference			Reference		
52-63	1.28	(0.89-1.85)	0.180	1.22	(0.85-1.77)	0.29
>=64	2.52	(1.74-3.63)	<0.001	2.36	(1.62-3.45)	<0.001
Sex						
Female	Reference					
Male	0.96	(0.69-1.34)	0.821			
Histologic type						
NKC	Reference					
BSCC	1.01	(0.45-2.29)	0.974			
T stage						
T4	Reference			Reference		
T1	0.68	(0.45-1.02)	0.064	0.62	(0.40-0.96)	0.03
T2	0.66	(0.41-1.06)	0.084	0.63	(0.38-1.03)	0.07
T3	0.59	(0.40-0.87)	0.008	0.64	(0.43-0.95)	0.03
N stage						
N3	Reference			Reference		
N0	0.98	(0.59-1.63)	0.947	0.77	(0.44-1.34)	0.35
N1	0.68	(0.43-1.07)	0.096	0.57	(0.34-0.94)	0.03
N2	0.68	(0.46-1.00)	0.051	0.68	(0.46-1.00)	0.05
Clinical stage						
IVB	Reference					
III	0.56	(0.38-0.83)	0.004			
IVA	1	(0.68-1.47)	0.994			
Chemotherapy						
No	Reference			Reference		
Yes	0.6	(0.33-1.11)	0.103	0.86	(0.46-1.63)	0.65

HR, hazard ratio; CI, confidence interval; NKC, nonkeratinizing squamous cell carcinoma; BSCC, basaloid squamous cell carcinoma; T, tumor; N node.

### 3.3 Nomogram details

**Figure 2:**
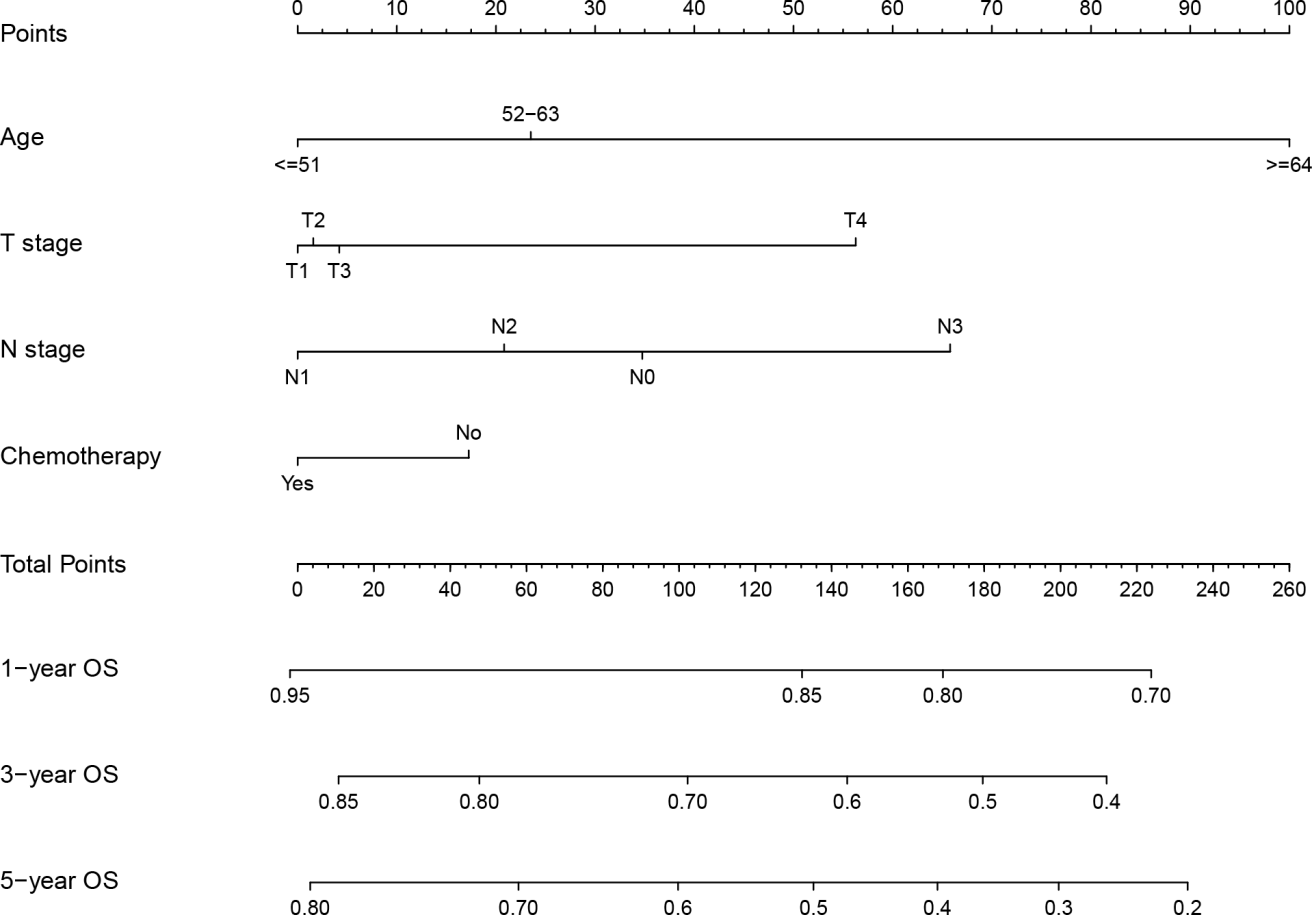
Nomogram for prediction of 1-, 3- and 5-year overall survival of locoregionally advanced nasopharyngeal carcinoma.

The above prognostic variables were included in our nomogram for OS based on the multivariate Cox analysis (**Figure 2**). The probability of the 3- and 5-year OS for an individual could be predicted through this nomogram. In the nomogram for each patient, each parameter axis has a value, and a straight line can be drawn upward to the corresponding points axis. After adding up the scores, the sum is marked on the total points axis and a line drawn downward to the survival axis gives the patient’s predicted OS. For example, a 60-year-old (23.5 points) LA-NPC patient at TNM stage T3 (4.2 points), N2 (20.8 points) M0, who received radiotherapy and chemotherapy (0 points) is assigned 62.3 total points and the estimated probability of 3- and 5-year OS is 79.0% and 72.0%, respectively, according to the nomogram.

### 3.4 Nomogram discrimination

**Figure 3:**
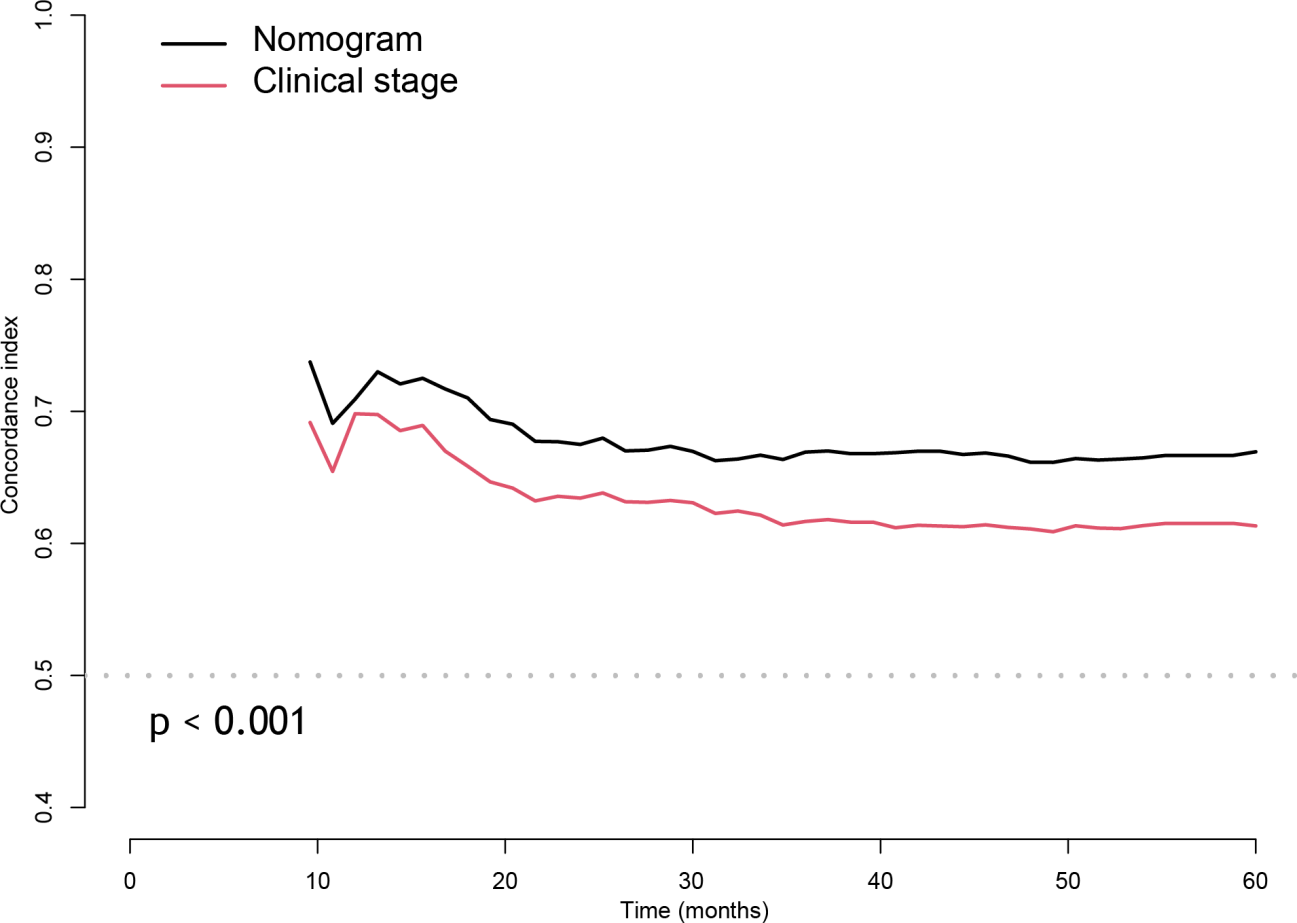
The Concordance index of nomogram compared with the clinical stage with cross-validation in external validation cohort.

**Figure 4:**
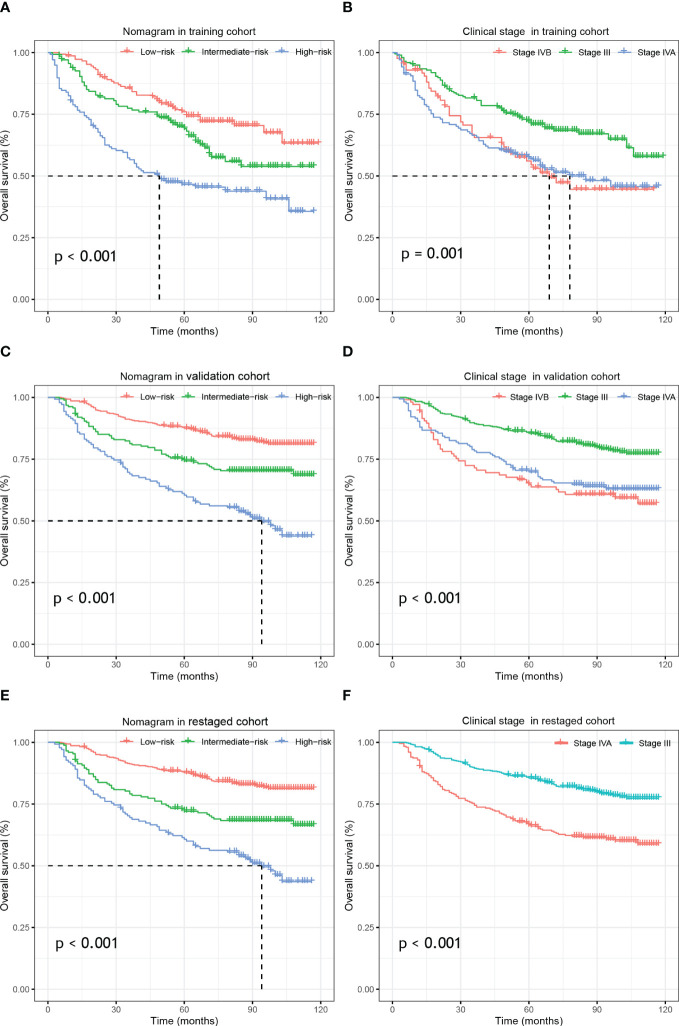
Kaplan–Meier survival analysis of overall survival according to the nomogram(left) and the clinical stage (right) stratification in the training cohort **(A, B)**, external validation cohort **(C, D)** and re-staged cohort **(E, F)**.

In the training cohort, the bias-corrected C-indices for the nomogram and clinical stage were 0.63 (95%CI: 0.61-0.69) and 0.57 (95%CI: 0.53-0.61), respectively. In the external validation cohort, based on the 7^th^ edition of the AJCC staging system, the C-index of the nomogram was still higher than that of the clinical stage, being 0.67 (95%CI: 0.64-0.71) versus 0.60 (95%CI: 0.57-0.64), respectively (**Figure 3**). After restaging based on the 8^th^ edition, the C-indices of the nomogram and clinical stage were 0.68 (95%CI: 0.64-0.71) and 0.60 (95%CI: 0.57-0.64), respectively, almost identical to the values based on the 7^th^ edition. The C-indices of the nomogram are always significantly higher compared to those of the clinical stage in three cohorts with p-values all <0.001. Furthermore, the *p*-values showed no significant difference between using the 7^th^ and the 8^th^ editions (0.94 for nomograms and 0.82 for the clinical stage). In the training cohort, we stratified patients into three groups on the basis of the cut-off values of the risk scores determined by the multivariate Cox analysis: a low-risk group (≤48.5, n=148), an intermediate-risk group (> 48.5 and < 93.5, n=136), and a high-risk group (≥93.5, n=137). In the training cohort, we also observed significant differences for OS rates among the low-risk, intermediate-risk and high-risk groups, with the 5-year OS rates being 76.0%, 69.2%, and 46.6%, respectively. Likewise, in the validation cohort, there were significant differences among the three risk groups, with the 5-year OS rates being 87.5% (n=433), 74.3% (n=188) and 60.4% (n=142), respectively, based on the 7^th^ version of the TNM staging system, or 87.7% (n=447), 72.1% (n=173) and 60.7% (n=138), respectively, after restaging based on the 8^th^ edition. The 5-year OS of the high-risk patients was significantly worse than that of the intermediate or low-risk group. Kaplan–Meier curves between the different groups showed greater separation with the nomogram than those based on clinical stages in both the training and validation cohorts (**Figure 4**). The high-risk group identified by the nomogram had a median survival of 94 months in both validation cohorts, whereas those groups classified by the clinical stage did not reach a median survival. In summary, the nomogram can always separate the high-risk group survival curve away from the others with lower median survival time and a significant p-value while the clinical stage cannot, which means the nomogram had greater ability to discriminate the prognosis outcomes for the low-risk, intermediate-risk and high-risk subgroups, as compared to the traditional staging system.

### 3.5 Nomogram calibration and DCA

**Figure 5:**
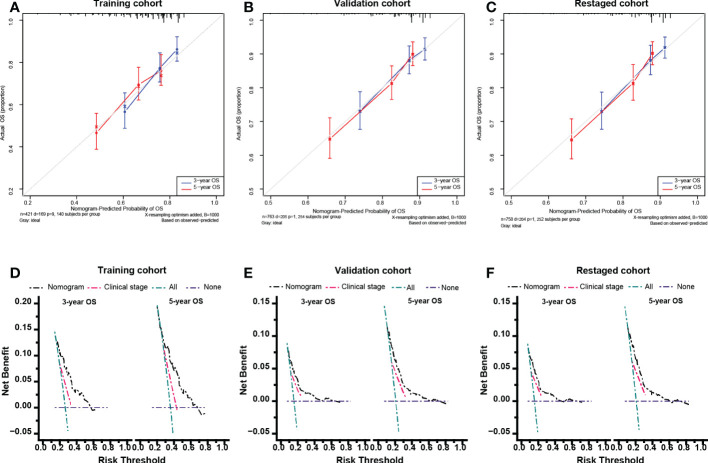
Calibration curves and Decision curve analysis. Calibration curves for 5-year overall survival in the training cohort **(A)**, validation cohort **(B)** and re-staged cohort **(C)**. Decision curve analysis for 5-year overall survival in the training cohort **(D)**, validation cohort **(E)** and re-staged cohort **(F)**.

The calibration curve in **Figure 5** nearly coincided with the actual survival, indicating that the model is well calibrated. The consequences of DCA illustrate that our nomogram is of higher clinical net benefit than traditional clinical staging for forecasting OS.

### 3.6 Subgroup analysis

**Figure 6:**
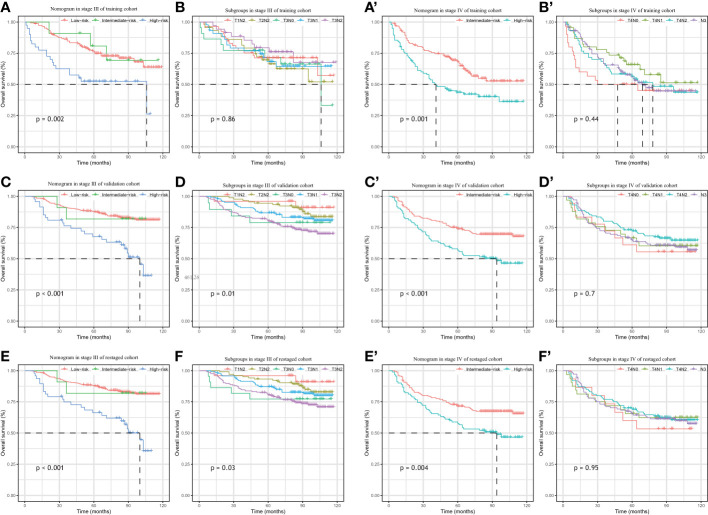
Kaplan–Meier survival analysis of overall survival according to the nomogram(left) and the clinical stage subgroup (right) stratification in the patients with stage III & stage IV from training cohort (**A**, **B**, **A’, B’**), external validation cohort (**C**, **D C’, D’**) and re-staged cohort (**E**, **F**, **E’, F’**).

As shown in the Kaplan–Meier curves, the nomogram was easily able to identify the high-risk LA-NPC patients in stage III and stage IV, with 5-year OS rates 67.7% and 56.8%, respectively. As shown in the **Figure 6**, the high-risk group survival curves identified by the nomogram are always separated from the others with significantly lower median survival time (p-value < 0.01) while the curves of the subgroups crossed, failing to stratify the risk of the patients. Therefore, our nomogram had better discriminative ability than subgroups.

### 3.7 Independent factors for the high-risk group

**Figure 7:**
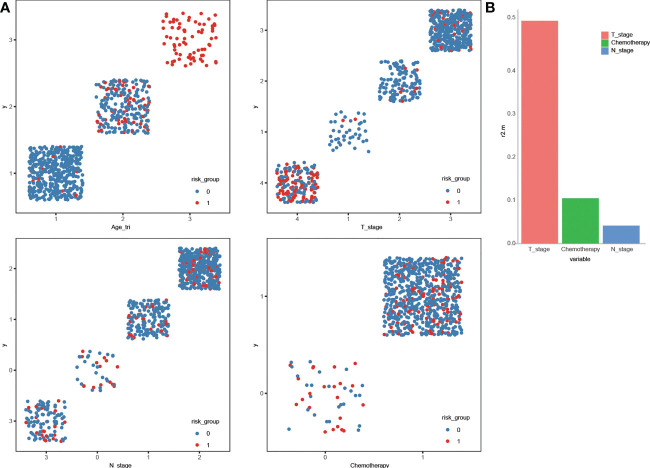
Scatter plot displaying the stratification of high-risk and non- high-risk by the variables **(A)**. General dominance of independent predictors for becoming high-risk in locoregionally advanced nasopharyngeal carcinoma **(B)**.

Scatter plots in **Figure 7** give obvious visual cues about how the high-risk patients and non-high-risk patients are clustered or varies in different categories. Elderly patients at advanced stages and without chemotherapy were more prone to have high risk than the other patients. Patients at or above a certain age (≥64) were uniformly identified as high-risk patients and this complete separation age leads to a warning when we were performing the multivariate logistic regression analysis in R software. Age was the most crucial determinant for high risk, and dominance analysis demonstrated that T stage was second in importance (average contribution, 0.52), followed by chemotherapy (0.10) and N stage (0.04) (**Figure 7**).

## 4 Discussion

To best of our knowledge, this is the first OS predictive nomogram for LA-NPC patients built from the SEER database and validated with a large sample institutionally. In this retrospective cohort study, we developed a novel prognostic tool based on clinical risk factors from the SEER database and showed an improved the ability to predict OS in LA-NPC using external data in our own center. Results show that our model can categorize patients into high-risk, intermediate-risk and low-risk groups with significantly different OS, which the current TNM staging system cannot do. Furthermore, our classifier performed significantly better than the subgroups at risk stratification. Our verified nomogram is a simple and accurate method for predicting OS in LA-NPC patients and provides new options for individualized treatment which complement the traditional and time-honored TNM staging system. To those high-risk patients identified by the nomogram, their poorer outcomes challenge the consistent treatment put forward by the NCCN guidelines, and the treatment intensity deserves to be carefully weighed.

SEER is an authoritative source for cancer statistics in the United States where the incidence is low but WHO type I is observed as the predominant histological type. However, type I is so uncommon in endemic areas. In the south China, patients histologically diagnosed with WHO type II and type III NPC accounts for greater than 95% of all NPC (1, 36, 37), and are radioresponsive and associated with Epstein-Barr virus (EBV) infection, and have better prognosis (1, 38). Therefore, we excluded the type II during the model construction, making it more generalizable to NPC patients in epidemic areas, including those from our institution.

Apart from the heterogeneous prognoses among the locoregionally advanced NPC, the current National Comprehensive Cancer Network (NCCN) Guideline recommends the same treatment for the different patient groups (CCRT + AC and IC + CCRT (2A) and CCRT (2B)) (39) for all stage II-IVA NPC. This catch−all strategy treats LA-NPC patients equally without discrimination and limits the clinicians’ access to prescribe personalized treatments. Recently, CSCO and ASCO issued joint guidelines for evidence-based stratified recommendations, in which de-intensified treatment was recommended for stage II and T3N0 patients (RT alone for T2N0, CCRT for T2N1 and T3N0) (40). A relatively lower risk has been reported in patients with T3N0 NPC compared to other LA-NPC patients due to lack of nodal involvement (41). N0 patients have therefore often been excluded from clinical trials and other studies when it came to locally regional disease (13, 42–44). However, as shown by our Kaplan–Meier survival curves (**Figure 6**), patients with T3-4N0 did not display better outcomes than the other groups, which could be due to the small number of the patients. Additional research is needed to analyze the prognosis of negative nodal metastasis LA-NPC patients.

Our data shows that in our nomogram age is the most dominant prognostic factor. In several previous studies based on the SEER database, the survival of older NPC patients has been reported to be inferior to that of the younger patients (24, 26), which was also verified by Yan et al. in their own institution (23). Consistent with the findings from SEER, data from the China National Mortality Surveillance System (NMSS) demonstrated that mortality and years of life lost (YLL) due to NPC increases with age (45). Age always appears as one of the significant parameters in the published NPC nomograms combined with other variables (7, 8, 12, 14, 46). Advanced age is a major impediment for oncologists to prescribe the guideline-recommended treatment. According a population-based analysis of 3880 NPC patients, younger patients were more likely to receive chemotherapy (26). On the other hand, elderly NPC patients have poorer tolerance to radiotherapy (47). Comorbidity has been found to be associated with increased overall mortality in elderly cancer patients, which may result from the delay of diagnosis, the ineligibility for radiotherapy and chemotherapy (48, 49). Therefore, the management of comorbidities must be taken into account in clinics.

Radiotherapy combined with chemotherapy has been the standard treatment for LA-NPC patients since the landmark Intergroup 0099 randomized trial (50, 51). In the era of intensity-modulated radiotherapy (IMRT), distant metastasis instead of locoregional recurrence has become the main cause of treatment failure (52). Consistent with our result, the use of chemotherapy was a protective factor for LA-NPC patients and patients without chemotherapy were more likely to be in the high-risk category. Xiang et al. analyzed 1452 patients from the SEER database and verified that chemotherapy offers a significant substantial survival benefit for stage III NPC patients (18). Luo et al. explored the efficacy of chemotherapy of 729 patients from the SEER database and indicated that chemoradiotherapy could improve the survival of IVA patients (16). However, research into the optimum chemotherapy strategies for LA-NPC patients in regard to sequence, dose, and cycle are still under study.

This study has some limitations. On one hand, we ensured the consistency of the histological types of the cohorts at the expense of using a larger sample size from SEER. On the other hand, due to the inevitable limitations of the SEER database, we could not obtain some important information, such as plasma Epstein-Barr virus (EBV) DNA, chemotherapy regimens, radiotherapy details, and comorbidity status. All in all, our nomogram remains to be fully verified in more data centers and it can be improved with more information based on a larger sample size or our center data for LA-NPC prognosis prediction. In this way, we can target high-risk patients more precisely.

In conclusion, we built an OS predictive nomogram that can help clinicians better predict the prognosis of LA-NPC patients, compared with the traditional tumor-node-metastasis staging method, and identify those at high risk for more personalized treatment.

## Data availability statement

The original contributions presented in the study are included in the article/supplementary material. Further inquiries can be directed to the corresponding authors.

## Author contributions

YL and JC contributed to conception and design of the study. XW, SC, YH and YY organized the database. JC performed the statistical analysis. YL wrote the first draft of the manuscript. ZL and ZY were involved in manuscript revision. All authors contributed to the article and approved the submitted version.

## References

[1] Chen YP Chan ATC Le QT Blanchard P Sun Y Ma J. Nasopharyngeal carcinoma. Lancet (Lond Engl) (2019) 394(10192):64–80. doi: 10.1016/S0140-6736(19)30956-0 31178151

[2] Sung H Ferlay J Siegel RL Laversanne M Soerjomataram I Jemal A. Global cancer statistics 2020: GLOBOCAN estimates of incidence and mortality worldwide for 36 cancers in 185 countries. CA: Cancer J Clin (2021) 71(3):209–49. doi: 10.3322/caac.21660 33538338

[3] Pan JJ Ng WT Zong JF Lee SW Choi HC Chan LL. Prognostic nomogram for refining the prognostication of the proposed 8th edition of the AJCC/UICC staging system for nasopharyngeal cancer in the era of intensity-modulated radiotherapy. Cancer (2016) 122(21):3307–15. doi: 10.1002/cncr.30198 PMC5524130 27434142

[4] Pan JJ Ng WT Zong JF Chan LL O’Sullivan B Lin SJ. Proposal for the 8th edition of the AJCC/UICC staging system for nasopharyngeal cancer in the era of intensity-modulated radiotherapy. Cancer (2016) 122(4):546–58. doi: 10.1002/cncr.29795 PMC4968037 26588425

[5] Hui EP Leung SF Au JS Zee B Tung S Chua D. Lung metastasis alone in nasopharyngeal carcinoma: A relatively favorable prognostic group. A study by the Hong Kong nasopharyngeal carcinoma study group. Cancer (2004) 101(2):300–6. doi: 10.1002/cncr.20358 15241827

[6] Mao YP Xie FY Liu LZ Sun Y Li L Tang LL. Re-evaluation of 6th edition of AJCC staging system for nasopharyngeal carcinoma and proposed improvement based on magnetic resonance imaging. Int J Radiat Oncol Biol Phys (2009) 73(5):1326–34. doi: 10.1016/j.ijrobp.2008.07.062 19153016

[7] Zhong L Dong D Fang X Zhang F Zhang N Zhang L. A deep learning-based radiomic nomogram for prognosis and treatment decision in advanced nasopharyngeal carcinoma: A multicentre study. EBioMedicine (2021) 70:103522. doi: 10.1016/j.ebiom.2021.103522 34391094 PMC8365370

[8] Zhao R Liang Z Chen K Zhu X. Nomogram based on inflammatory biomarkers and nutritional indicators for predicting overall survival in locoregionally advanced nasopharyngeal carcinoma. J Inflamm Res (2022) 15:2971–81. doi: 10.2147/JIR.S366299 PMC9122053 35602661

[9] Tang XR Li YQ Liang SB Jiang W Liu F Ge WX. Development and validation of a gene expression-based signature to predict distant metastasis in locoregionally advanced nasopharyngeal carcinoma: a retrospective, multicentre, cohort study. Lancet Oncol (2018) 19(3):382–93. doi: 10.1016/S1470-2045(18)30080-9 29428165

[10] OuYang PY You KY Zhang LN Xiao Y Zhang XM Xie FY. External validity of a prognostic nomogram for locoregionally advanced nasopharyngeal carcinoma based on the 8th edition of the AJCC/UICC staging system: A retrospective cohort study. Cancer Commun (Lond Engl) (2018) 38(1):55. doi: 10.1186/s40880-018-0324-x PMC6122160 30176932

[11] Liu SL Sun XS Chen QY Liu ZX Bian LJ Yuan L. Development and validation of a transcriptomics-based gene signature to predict distant metastasis and guide induction chemotherapy in locoregionally advanced nasopharyngeal carcinoma. Eur J Cancer (Oxf Engl: 1990) (2022) 163:26–34. doi: 10.1016/j.ejca.2021.12.017 35032814

[12] Liu LT Liang YJ Guo SS Mo HY Guo L Wen YF. Induction chemotherapy followed by radiotherapy versus concurrent chemoradiotherapy in the treatment of different risk locoregionally advanced nasopharyngeal carcinoma. Ther Adv Med Oncol (2020) 12:1758835920928214. doi: 10.1177/1758835920928214 32536983 PMC7268167

[13] Li WF Chen NY Zhang N Hu GQ Xie FY Sun Y. Concurrent chemoradiotherapy with/without induction chemotherapy in locoregionally advanced nasopharyngeal carcinoma: Long-term results of phase 3 randomized controlled trial. Int J Cancer (2019) 145(1):295–305. doi: 10.1002/ijc.32099 30613964

[14] He Y Yang D Zhou T Xue W Zhang J Li F. Epstein-Barr Virus DNA loads in the peripheral blood cells predict the survival of locoregionally-advanced nasopharyngeal carcinoma patients. Cancer Biol Med (2021) 18(3):888–99. doi: 10.20892/j.issn.2095-3941.2020.0464 PMC8330545 33960178

[15] Zhou ZR Wang WW Li Y Jin KR Wang XY Wang ZW. In-depth mining of clinical data: the construction of clinical prediction model with r. Ann Trans Med (2019) 7(23):796. doi: 10.21037/atm.2019.08.63 PMC6989986 32042812

[16] Luo HD Xia FJ Wu JH Yi B. Efficacy of chemoradiotherapy in survival of stage IV nasopharyngeal carcinoma and establishment of a prognostic model. Oral Oncol (2022) 131:105927. doi: 10.1016/j.oraloncology.2022.105927 35679694

[17] Huang ST Su DK. Survival among subgroups of patients with stage II nasopharyngeal carcinoma. Sci Rep (2022) 12(1):7007. doi: 10.1038/s41598-022-11145-4 35488053 PMC9054756

[18] Xiang ZF Hu DF Xiong HC Li MY Zhang ZC Shen ED. Benefit of chemotherapy in stage III nasopharyngeal carcinoma: Analysis of the surveillance, epidemiology, and end results database. Oral Oncol (2021) 117:105284. doi: 10.1016/j.oraloncology.2021.105284 33845238

[19] Chen H Huang Z Chen L Li Y Zhao T Wei Q. Characteristics of early death in patients with localized nasopharyngeal cancer: A population-based SEER analysis. Front Oncol (2021) 11:580220. doi: 10.3389/fonc.2021.580220 33791199 PMC8006381

[20] Piao Y Jiang C Yan F Ye Z Fu Z Jiang H. Therapeutic patterns and outcomes in older patients (aged ≥65 years) with stage II-IVB nasopharyngeal carcinoma: an investigational study from SEER database. J Cancer (2020) 11(18):5273–80. doi: 10.7150/jca.46201 PMC7391202 32742473

[21] Li W Lu H Wang H Hu L Sun X Yu H. Establishment and validation of a novel nomogram to predict overall survival in nasopharyngeal carcinoma with lymph node metastasis. Head Neck (2021) 43(8):2353–63. doi: 10.1002/hed.26687 33780078

[22] Qu W Li S Zhang M Qiao Q. Pattern and prognosis of distant metastases in nasopharyngeal carcinoma: A large-population retrospective analysis. Cancer Med (2020) 9(17):6147–58. doi: 10.1002/cam4.3301 PMC7476823 32649056

[23] Yan C Tu Z Zhang Z Ouyang X Li D Peng S. Institutionally validated nomogram predicting prognosis for older patients with nonmetastatic nasopharyngeal carcinoma. Future Oncol (Lond Engl) (2022) 18(15):1829–38. doi: 10.2217/fon-2021-1121 35179075

[24] Huang SJ Tang YY Liu HM Tan GX Wang X Zhang H. Impact of age on survival of locoregional nasopharyngeal carcinoma: An analysis of the surveillance, epidemiology, and end results program database, 2004-2013. Clin Otolaryngol (2018) 43(5):1209–18. doi: 10.1111/coa.13124 29688619

[25] Huang Y Chen W Haque W Verma V Xing Y Teh BS. The impact of comorbidity on overall survival in elderly nasopharyngeal carcinoma patients: a national cancer data base analysis. Cancer Med (2018) 7(4):1093–101. doi: 10.1002/cam4.1377 PMC5911579 29493889

[26] Wu SG Liao XL He ZY Tang LY Chen XT Wang Y. Demographic and clinicopathological characteristics of nasopharyngeal carcinoma and survival outcomes according to age at diagnosis: A population-based analysis. Oral Oncol (2017) 73:83–7. doi: 10.1016/j.oraloncology.2017.08.006 28939081

[27] Vaughan TL Shapiro JA Burt RD Swanson GM Berwick M Lynch CF. Nasopharyngeal cancer in a low-risk population: defining risk factors by histological type. Cancer Epidemiol Biomarkers Prev (1996) 5(8):587–93. 8824359

[28] Guo R Wu H Wang J Lian CL He ZY Zhang WW. Lymph node status and outcomes for nasopharyngeal carcinoma according to histological subtypes: A SEER population-based retrospective analysis. Adv Ther (2019) 36(11):3123–33. doi: 10.1007/s12325-019-01100-7 31559602

[29] Pan XX Liu YJ Yang W Chen YF Tang WB Li CR. Histological subtype remains a prognostic factor for survival in nasopharyngeal carcinoma patients. Laryngoscope (2020) 130(3):E83–e8. doi: 10.1002/lary.28099 31188486

[30] Wang Q Xie H Li Y Theodoropoulos N Zhang Y Jiang C. Racial and ethnic disparities in nasopharyngeal cancer with an emphasis among Asian americans. Int J Cancer (2022) 151(8):1291–1303. doi: 10.1002/ijc.34154 35666524

[31] Wang Y Zhang Y Ma S. Racial differences in nasopharyngeal carcinoma in the united states. Cancer Epidemiol (2013) 37(6):793–802. doi: 10.1016/j.canep.2013.08.008 24035238 PMC3851929

[32] Sun LM Li CI Huang EY Vaughan TL. Survival differences by race in nasopharyngeal carcinoma. Am J Epidemiol (2007) 165(3):271–8. doi: 10.1093/aje/kwk008 17090616

[33] Camp RL Dolled-Filhart M Rimm DL. X-Tile: a new bio-informatics tool for biomarker assessment and outcome-based cut-point optimization. Clin Cancer Res (2004) 10(21):7252–9. doi: 10.1158/1078-0432.CCR-04-0713 15534099

[34] Moons KG Altman DG Reitsma JB Ioannidis JP Macaskill P Steyerberg EW. Transparent reporting of a multivariable prediction model for individual prognosis or diagnosis (TRIPOD): Explanation and elaboration. Ann Internal Med (2015) 162(1):W1–73. doi: 10.7326/M14-0698 25560730

[35] Vickers AJ Elkin EB. Decision curve analysis: a novel method for evaluating prediction models. Med Decision Making (2006) 26(6):565–74. doi: 10.1177/0272989X06295361 PMC2577036 17099194

[36] Marks JE Phillips JL Menck HR. The national cancer data base report on the relationship of race and national origin to the histology of nasopharyngeal carcinoma. Cancer (1998) 83(3):582–8. doi: 10.1002/(SICI)1097-0142(19980801)83:3<582::AID-CNCR29>3.0.CO;2-R 9690553

[37] Wang HY Chang YL To KF Hwang JS Mai HQ Feng YF. A new prognostic histopathologic classification of nasopharyngeal carcinoma. Chin J cancer (2016) 35:41. doi: 10.1186/s40880-016-0103-5 27146632 PMC4857443

[38] Pathmanathan R Prasad U Chandrika G Sadler R Flynn K Raab-Traub N. Undifferentiated, nonkeratinizing, and squamous cell carcinoma of the nasopharynx. variants of Epstein-Barr virus-infected neoplasia. Am J Phatol (1995) 146(6):1355–67. PMC1870892 7778675

[39] Pfister DG Spencer S Adelstein D Adkins D Anzai Y Brizel DM. Head and neck cancers, version 2.2020, NCCN clinical practice guidelines in oncology. J Natl Compr Cancer Netw: JNCCN (2020) 18(7):873–98. doi: 10.6004/jnccn.2020.0031 32634781

[40] Chen YP Ismaila N Chua MLK Colevas AD Haddad R Huang SH. Chemotherapy in combination with radiotherapy for definitive-intent treatment of stage II-IVA nasopharyngeal carcinoma: CSCO and ASCO guideline. J Clin Oncol (2021) 39(7):840–59. doi: 10.1200/JCO.20.03237 33405943

[41] Guo R Tang LL Mao YP Du XJ Chen L Zhang ZC. Proposed modifications and incorporation of plasma Epstein-Barr virus DNA improve the TNM staging system for Epstein-Barr virus-related nasopharyngeal carcinoma. Cancer (2019) 125(1):79–89. doi: 10.1002/cncr.31741 30351466

[42] Chen L Hu CS Chen XZ Hu GQ Cheng ZB Sun Y. Adjuvant chemotherapy in patients with locoregionally advanced nasopharyngeal carcinoma: Long-term results of a phase 3 multicentre randomised controlled trial. Eur J Cancer (Oxf Engl: 1990) (2017) 75:150–8. doi: 10.1016/j.ejca.2017.01.002 28235726

[43] Zhang Y Chen L Hu GQ Zhang N Zhu XD Yang KY. Gemcitabine and cisplatin induction chemotherapy in nasopharyngeal carcinoma. N Engl J Med (2019) 381(12):1124–35. doi: 10.1056/NEJMoa1905287 31150573

[44] Yang Q Cao SM Guo L Hua YJ Huang PY Zhang XL. Induction chemotherapy followed by concurrent chemoradiotherapy versus concurrent chemoradiotherapy alone in locoregionally advanced nasopharyngeal carcinoma: long-term results of a phase III multicentre randomised controlled trial. Eur J Cancer (Oxf Engl: 1990) (2019) 119:87–96. doi: 10.1016/j.ejca.2019.07.007 31425966

[45] Long Z Wang W Liu W Wang F Meng S Liu J. Trend of nasopharyngeal carcinoma mortality and years of life lost in China and its provinces from 2005 to 2020. Int J Cancer (2022) 151(5):684–91. doi: 10.1002/ijc.33998 35285029

[46] Tang LQ Li CF Li J Chen WH Chen QY Yuan LX. Establishment and validation of prognostic nomograms for endemic nasopharyngeal carcinoma. J Natl Cancer Institute (2016) 108(1). doi: 10.1093/jnci/djv291 26467665

[47] Sze HC Ng WT Chan OS Shum TC Chan LL Lee AW. Radical radiotherapy for nasopharyngeal carcinoma in elderly patients: the importance of co-morbidity assessment. Oral Oncol (2012) 48(2):162–7. doi: 10.1016/j.oraloncology.2011.08.019 21925925

[48] Lee L Cheung WY Atkinson E Krzyzanowska MK. Impact of comorbidity on chemotherapy use and outcomes in solid tumors: A systematic review. J Clin Oncol (2011) 29(1):106–17. doi: 10.1200/JCO.2010.31.3049 21098314

[49] Jørgensen TL Hallas J Friis S Herrstedt J. Comorbidity in elderly cancer patients in relation to overall and cancer-specific mortality. Br J Cancer (2012) 106(7):1353–60. doi: 10.1038/bjc.2012.46 PMC3314782 22353805

[50] Al-Sarraf M LeBlanc M Giri PG Fu KK Cooper J Vuong T. Chemoradiotherapy versus radiotherapy in patients with advanced nasopharyngeal cancer: phase III randomized intergroup study 0099. J Clin Oncol (1998) 16(4):1310–7. doi: 10.1200/JCO.1998.16.4.1310 9552031

[51] Blanchard P Lee A Marguet S Leclercq J Ng WT Ma J. Chemotherapy and radiotherapy in nasopharyngeal carcinoma: An update of the MAC-NPC meta-analysis. Lancet Oncol (2015) 16(6):645–55. doi: 10.1016/S1470-2045(15)70126-9 25957714

[52] Lai SZ Li WF Chen L Luo W Chen YY Liu LZ. How does intensity-modulated radiotherapy versus conventional two-dimensional radiotherapy influence the treatment results in nasopharyngeal carcinoma patients? Int J Radiat Oncol Biol Phys (2011) 80(3):661–8. doi: 10.1016/j.ijrobp.2010.03.024 20643517

